# Supplementary material for: Causal Effects of Motor Control on Gait Kinematics After Orthopedic Surgery in Cerebral Palsy: A Machine-Learning Approach
Source: Front Hum Neurosci. 2022 Jun 3;16:846205. doi: 10.3389/fnhum.2022.846205 (PMC9204855; doi:10.3389/fnhum.2022.846205)
Supplement: Supplementary file 1 [file Data_Sheet_1.pdf]

Supplementary Material

1 Directed Acyclic Graph

In this study we evaluate the impact of SEMLS (exposure) on ΔGDI (outcome).

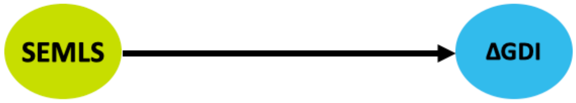

The decision to have a SEMLS does not directly cause a change in gait kinematics. Rather, ΔGDI is mediated by a change in impairments (ΔImp) caused by the surgery. Since ΔImp is a mediator between SEMLS and ΔGDI, it does not appear in the adjustment set to evaluate the impact of SEMLS on ΔGDI. Including measures of ΔImp in the BART models would introduce bias.

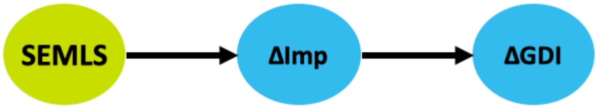

Baseline age and impairment level (Imp_{pre}) have a causal effect on these factors. Age also effects Imp_{pre} and GDI_{pre}. Baseline impairment also has a causal effect on GDI_{pre}. Baseline GDI and ΔGDI are related by measurement technique (*i.e.,* noise and error, regression to the mean) and other unmeasured variables not included in the DAG, as shown by the bidirectional arrow.

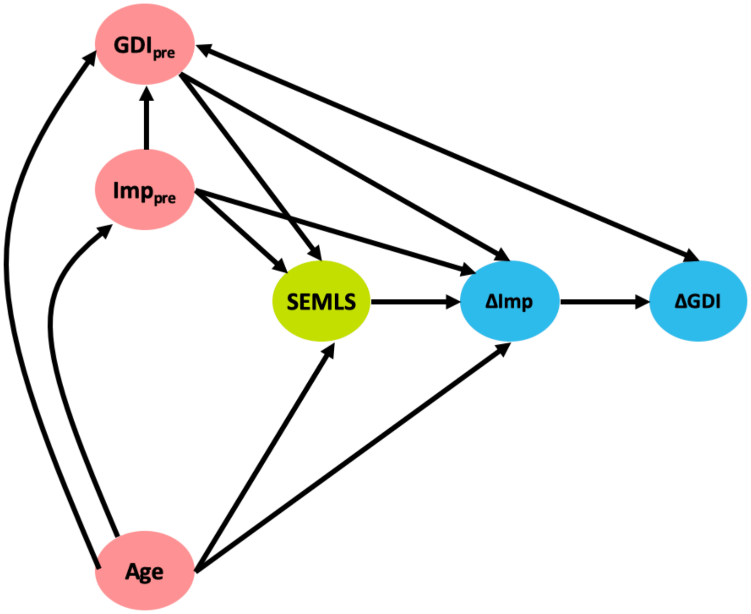

Prior surgical history (Hx) also impacts the decision to undergo SEMLS and baseline impairment levels. We also recognize that there are unmeasured factors related to an individual's impairment severity (Sev) that impact their history and baseline impairment. Note that severity was not specified in the final adjustment set, indicating that adjusting for severity is not necessary to evaluate the impact of SEMLS or impairments on ΔGDI.

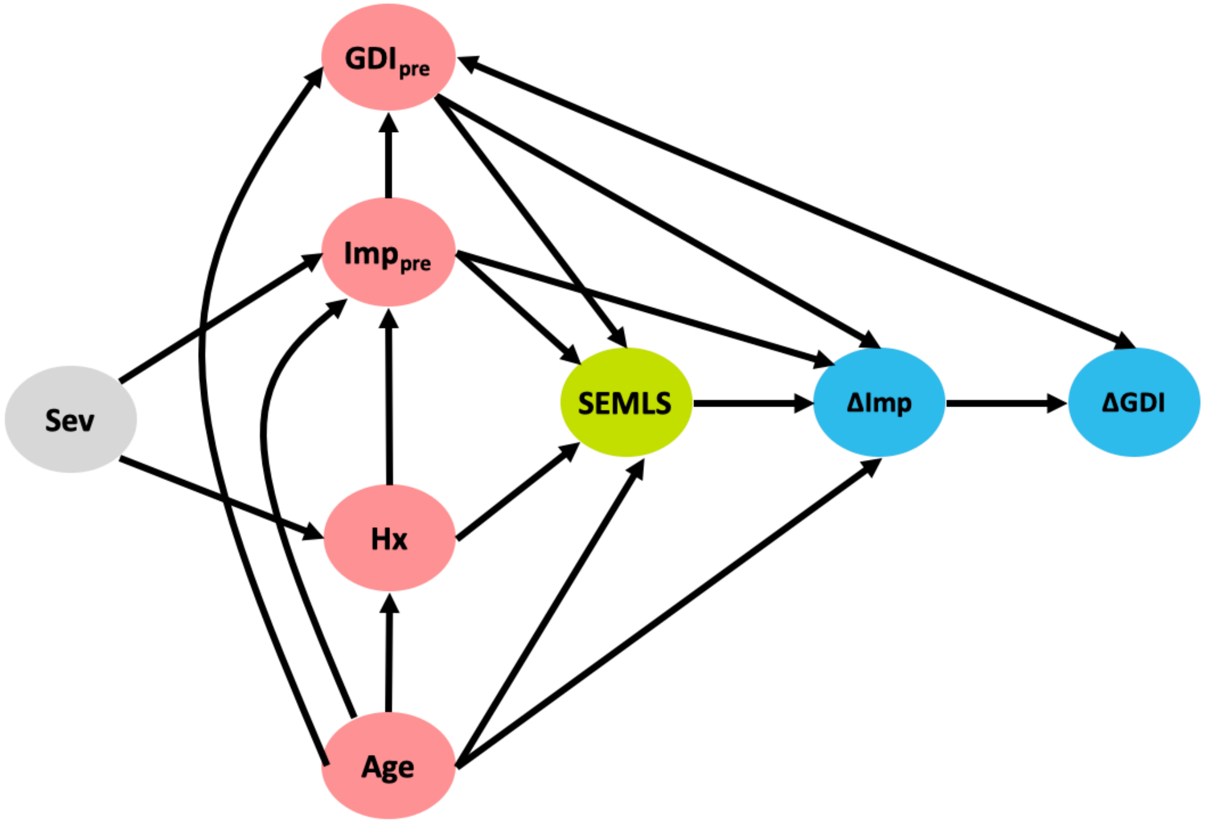

2 Missing Data

Using data collected as part of clinical care often means that values are missing for a given visit. We used the R Multivariate Imputation by Chained Equations Package (MICE), which uses Fully Conditional Specification, a method where a separate model is developed for each variable to impute missing data.

Table 1: Number of participants missing each study variable

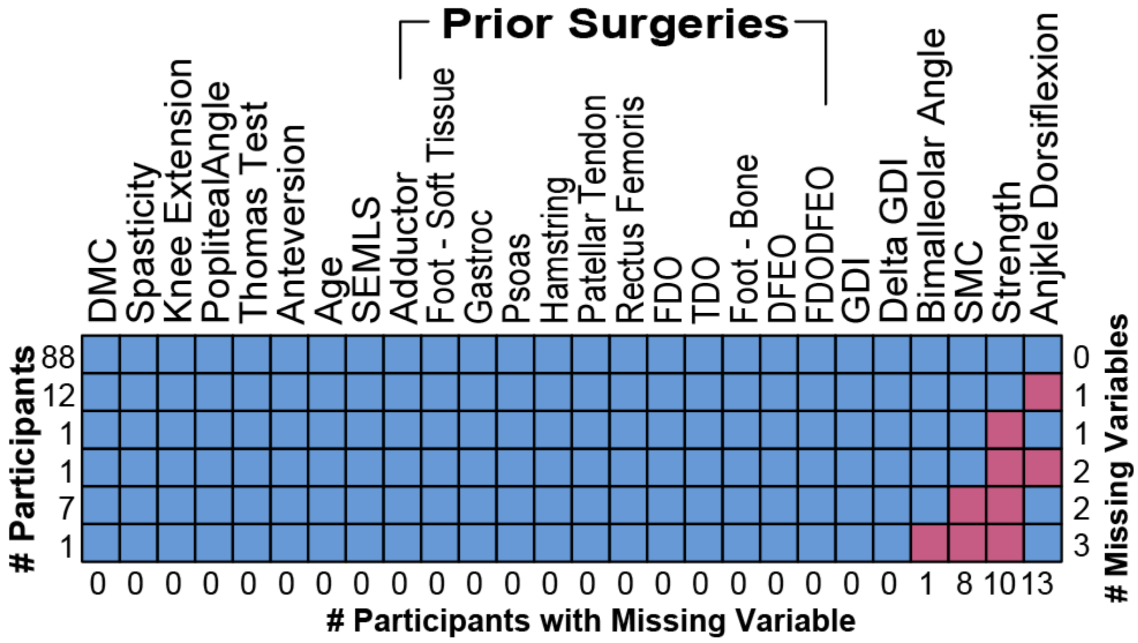

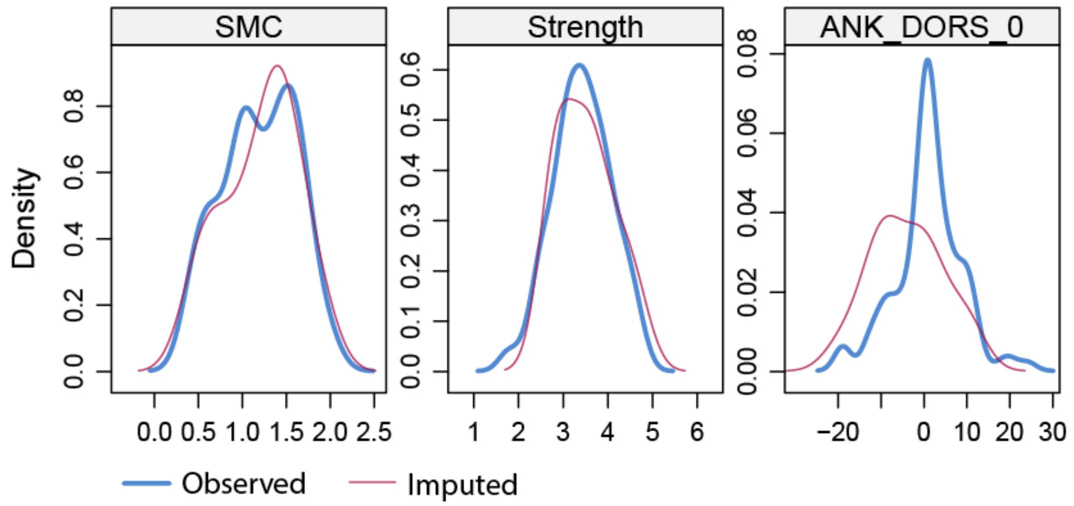

Figure 1: Density plots of study variables that had missing values. The blue line shows the observed distribution of each variable among participants with available data, and the red line shows the distribution of imputed missing data. SMC: Selective Motor Control.

3 Implied Conditional Independencies

We evaluated the implied conditional independencies specified from the assumed causal relationships defined in our DAG. All partial correlations were less than 0.30, supporting model plausibility.

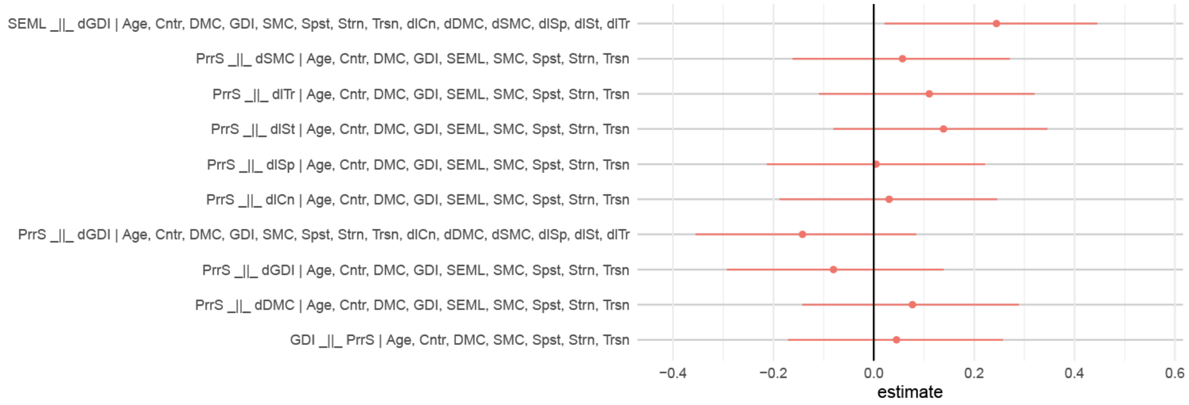

4 Change in Impairments between Visits

	**No SEMLS**		**SEMLS**	
	Pre	***Post***	***Pre***	***Post***
**GDI**	69.4 (10.0)	69.2 (11.9)	68.8 (12.0)	71.5 (11.7)
**SMC**	1.24 (0.42)	1.31 (0.44)	1.11 (0.40)	1.29 (0.47)
**DMC**	81.1 (9.0)	80.3 (9.0)	80.5 (9.5)	77.8 (8.6)
**Strength**	3.37 (0.59)	3.55 (0.68)	3.52 (0.63)	3.56 (0.67)
**Spasticity**	1.16 (0.42)	1.15 (0.37)	1.29 (0.46)	1.20 (0.37)
**Anteversion (°)**	36.3 (10.4)	35.8 (11.2)	39.8 (11.3)	17.9 (7.7)
**Bimalleolar (°)**	12.8 (10.6)	13.5 (10.3)	13.4 (11.3)	14.0 (6.8)
**Dorsiflexion (°)**	0.32 (8.52)	0.36 (8.55)	-0.96 (7.63)	3.89 (6.36)
**Knee Extension (°)**	0.52 (6.60)	1.51 (7.64)	0.12 (7.47)	-0.56 (5.95)
**Thomas Test (°)**	0.61 (6.23)	-0.61 (6.18)	2.21 (6.11)	-1.59 (4.32)
**Popliteal Angle (°)**	51.5 (15.4)	56.6 (15.0)	55.7 (12.8)	54.6 (10.9)
